# High Expression of EIF4G2 Mediated by the TUG1/Hsa-miR-26a-5p Axis Is Associated with Poor Prognosis and Immune Infiltration of Gastric Cancer

**DOI:** 10.1155/2022/9342283

**Published:** 2022-09-16

**Authors:** Liu Fu, Zhe Wang, Fengxiang Jiang, Guohua Wei, Longe Sun, Chuanyong Guo, Jianye Wu, Jianhuan Zhu

**Affiliations:** ^1^Department of Gastroenterology, Putuo People's Hospital, Tongji University, Shanghai 200060, China; ^2^Department of Gastroenterology, Tongji Hospital, Tongji University School of Medicine, Shanghai 200065, China; ^3^Department of Gastroenterology, Shanghai Tenth People's Hospital, Tongji University, Shanghai 200072, China

## Abstract

**Objective:**

Eukaryotic translation initiation factor 4 gamma 2 (EIF4G2) is involved in the occurrence and development of various tumors. However, the effect of EIF4G2 in gastric cancer (GC) has not been fully explored. The purpose of this study was to explore the function and mechanism of EIF4G2 in GC.

**Methods:**

The Tumor Immune Estimation Resource 2.0 database was used to analyze EIF4G2 expression in various cancers and the relationship between EIF4G2 expression and tumor-infiltrating immune cells. Gene Expression Profiling Interactive Analysis was utilized to assess the EIF4G2 expression level and its effect on survival in GC. UALCAN was conducted to analyze EIF4G2 expression in various subgroups of GC. The Kaplan–Meier plotter was employed for survival analysis. Receiver operator characteristic (ROC) curve analysis was applied to evaluate the diagnostic role of EIF4G2 in GC. LinkedOmics was used to identify the co-expressed genes and Gene Ontology and Kyoto Encyclopedia of Genes and Genomes pathways. The Tumor-Immune System Interaction database was employed to analyze the correlation between EIF4G2 expression and tumor-infiltrating lymphocytes. The starBase web platform was used to predict the upstream microRNAs and long noncoding RNAs.

**Results:**

EIF4G2 expression was upregulated in GC tissues compared to normal controls. High expression of EIF4G2 indicated poor prognosis in GC. ROC analysis revealed that EIF4G2 had good diagnostic ability to distinguish GC from normal tissues. Immune infiltration analysis indicated that EIF4G2 expression may be involved in the modulation of tumor immune infiltration in GC. Finally, we determined that the Taurine Upregulated 1 (TUG1)/hsa-miR-26a-5p/EIF4G2 axis was the most likely regulatory pathway involved in GC development.

**Conclusions:**

EIF4G2 was upregulated in GC and elevated expression of EIF4G2 indicated unfavorable prognosis. Moreover, EIF4G2 expression may be involved in the regulation of tumor immune cell infiltration. The TUG1/hsa-miR-26a-5p axis is a likely upstream regulatory mechanism of EIF4G2 in GC. EIF4G2 may thus serve as a prognosis biomarker and present a new therapeutic target.

## 1. Introduction

Gastric cancer (GC) is a serious threat to human health worldwide, which ranks fifth in incidence and fourth in mortality among all cancers [1, 2]. Since the early clinical symptoms are not obvious, most GC patients are already in the advanced stage when diagnosed [3]. Therapeutic methods are few and their effectiveness is limited to later-period tumor patients, when the 5-year survival rate is poor [4, 5]. Therefore, it is necessary to explore the molecular mechanisms that regulate GC occurrence and develop novel biomarkers for early diagnosis.

The function of eukaryotic translation initiation factors (eIFs) in tumorigenesis has been widely studied. Studies have shown that eIFs are highly expressed in many malignant tumors [6–8]. Eukaryotic translation initiation factor 4 gamma 2 (EIF4G2) is a member of the eIF family, which plays a vital role in regulating protein translation [9–11]. Research has revealed that abnormal expression of EIF4G2 is closely related to the occurrence and development of tumors. Some research has suggested EIF4G2 acts as oncogene. For example, downregulation of EIF4G2 by microRNA (miR)-379 inhibits the proliferation, migration and invasion of osteosarcoma cells [12]. Diffuse large B cell lymphoma (DLBCL) cell lines and patient samples have shown upregulated EIF4G2 expression, while downregulation of EIF4G2 decreased translation, cell proliferation and cell colony formation, thus inhibiting DLBCL development [13]. However, another study found that EIF4G2 transcripts were downregulated in bladder tumors, and this downregulation was associated with invasive tumors [14]. In GC, the effect of EIF4G2 has not been fully explored.

In the present study, various public online databases were utilized to analyze EIF4G2 expression. Firstly, Tumor Immune Estimation Resource 2.0 (TIMER2.0), Gene Expression Profiling Interactive Analysis (GEPIA), UALCAN databases, and the Kaplan–Meier plotter were employed to analyze the expression level and prognosis of EIF4G2 in GC. Then, the co-expression of genes and their relationship with immune cells were explored using LinkedOmics, TIMER2.0, and the Tumor-Immune System Interaction database (TISIDB). Finally, the starBase web platform was used to predict upstream microRNAs (miRNAs) and long non-coding RNAs (lncRNAs). In our study, we found that EIF4G2 was upregulated in GC, and high expression of EIF4G2 indicated poor prognosis. Moreover, EIF4G2 participated in the regulation of tumor immune cell infiltration in GC. The Taurine Upregulated 1 (TUG1)/hsa-miR-26a-5p axis was determined to be the most likely upstream regulatory mechanism for EIF4G2 in GC.

## 2. Methods

### 2.1. TIMER2.0 Database

TIMER2.0 (http://timer.cistrome.org/) is a web server for the systematic analysis of tumor-infiltrating immune cells (TIICs) across diverse cancer types [15]. It allows users to compare the expression of a gene between tumor and normal tissues in multiple cancers. In this study, the TIMER2.0 website was used to analyze differential expression of EIF4G2 in tumor and normal tissues in various cancers. We then predicted the relationship between EIF4G2 expression in GC and six tumor-infiltrating immune cells, including B cells, CD4+ T cells, CD8+ T cells, neutrophils, macrophages, and dendritic cells (DCs). In addition, we analyzed the correlation between the molecular markers of immune cells and expression of EIF4G2.

### 2.2. GEPIA Database

GEPIA (http://gepia.cancer-pku.cn/) is an interactive online web tool for the analysis of tissue samples from the cancer genome atlas (TCGA) and the genotype-tissue expression (GTEx) projects [16]. In this study, the website was used to explore mRNA expression of EIF4G2 in tumor and normal tissues. Also, we determined the association between EIF4G2 expression and overall survival of patients with GC.

### 2.3. UALCAN Database

UALCAN (http://ualcan.path.uab.edu/) is a comprehensive web portal to perform in-depth analyses of TCGA gene expression data [17]. In this study, UALCAN was employed to explore the mRNA expression of EIF4G2 across tumor and normal samples, as well as in various tumor subgroups based on patients' gender, nodal metastasis status, individual cancer stage, and tumor grade.

### 2.4. Kaplan–Meier Plotter (KM Plotter) Database

The KM plotter (http://kmplot.com/analysis/index.php?p=background) is a meta-analysis-based platform for survival analysis of 54 k genes (mRNA, miRNA, and protein-coding) in 21 types of cancers, the data sources of which include the gene expression omnibus (GEO), European Genome-phenome Archive, and TCGA [18]. In this study, the KM plotter was applied to evaluate the relationship between clinical outcomes and EIF4G2 expression in GC. The association between has-miR-26a expression and cancer survival was also evaluated using this web database.

### 2.5. Receiver Operating Characteristic (ROC) Curve

The diagnostic role of EIF4G2 in GC was evaluated by ROC curve analysis based on RNA sequencing (RNA-Seq) data in the transcripts per million (TPM) format of TCGA-GTEx, which were downloaded from UCSC XENA (https://xenabrowser.net/datapages/). R software (version 3.6.3) was used for analysis and visualization.

### 2.6. LinkedOmics Database

LinkedOmics (http://www.linkedomics.org/login.php) is a publicly available portal that includes multiomics data from all 32 TCGA cancer types and ten clinical proteomics tumor analysis consortium cancer cohorts [19]. In this study, the LinkFinder module of LinkedOmics was used to identify the co-expressed genes of EIF4G2 in GC and produce volcano plots and related heat maps. The co-expressed genes were then used for Gene Ontology (GO) and Kyoto Encyclopedia of Genes and Genomes (KEGG) pathway analysis in the LinkInterpreter module.

### 2.7. TISIDB Database

TISIDB (http://cis.hku.hk/TISIDB/) is an online web portal for examining tumor and immune system interactions [20]. In this study, we use the TISIDB to analyze the correlation between EIF4G2 expression and tumor-infiltrating lymphocytes (TILs) across human cancers. Based on the gene expression profile, the relative abundance of TILs was inferred by using gene set variation analysis. The correlations between EIF4G2 and TILs were measured by Spearman's test.

### 2.8. starBase Database Analysis

starBase(https://starbase.sysu.edu.cn/) is an open-source platform for exploring the miRNA-RNA, RNA-RNA, and protein-RNA interactions from UV cross-linking and immunoprecipitation (CLIP)-seq, degradome-seq, and RNA-RNA interactome data [21]. In this study, the online tool was used to predict miRNAs binding to EIF4G2. The miRNAs that were identified by more than two programs (PITA, RNA22, miRmap, microT, miRanda, PicTar, and TargetScan) were selected for subsequent analyses. The candidate lncRNAs were also explored by using the platform. In addition, starBase was employed to perform expression correlation analysis for miRNA-EIF4G2, lncRNA-miRNA, and lncRNA-EIF4G2 interactions in GC. The expression level of miRNAs and lncRNAs in tumor and normal samples was also analyzed in GC by starBase.

### 2.9. RNA-Seq Data of TUG1 in GC

The RNA-Seq data of TUG1 for expression level and survival analysis in GC were obtained from TCGA. For further study, RNA-Seq data with fragments per kilobase per million type format were converted to TPM format and subjected to log2 transformation. R software (version 3.6.3) was used for analysis and visualization.

### 2.10. Statistical Analysis

Student's *t*-test was used to compare the gene expression level in tumor and normal samples. Survival analysis was conducted using log-rank tests. ROC curve analysis was applied to detect the cutoff value of EIF4G2 using the pROC package (version 1.17.0.1). *P* < 0.05 was considered statistically significant in this study.

## 3. Results

### 3.1. EIF4G2 Was Upregulated in GC

**Figure 1:**
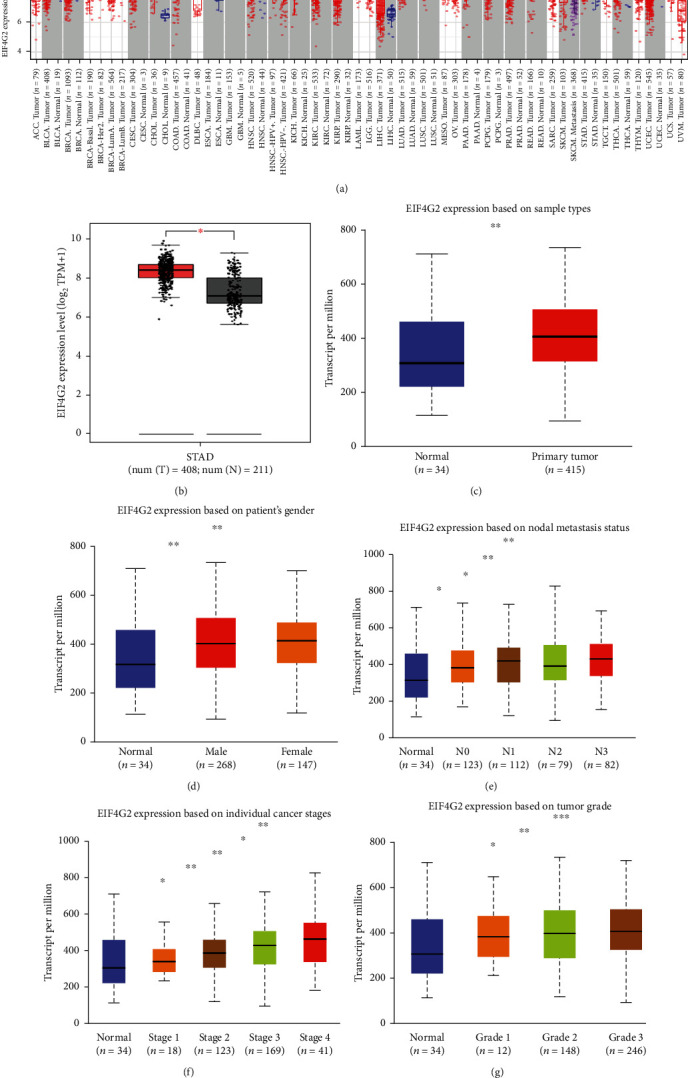
Expression analysis of EIF4G2 in GC using different online tools. (a) The expression of EIF4G2 in different types of tumor and normal tissues in the TIMER2.0 database. ^∗^*P* < 0.05, ^∗∗^*P* < 0.01, ^∗∗∗^*P* < 0.001. (b) EIF4G2 expression in TCGA GC tissues compared with corresponding TCGA and GTEx normal tissues (GEPIA). ^∗^*P* < 0.01. EIF4G2 expression level in GC (UALCAN) based on sample type (c), patient's gender (d), nodal metastasis status (e), individual cancer stage (f), and tumor grade (g). N0: no regional lymph node metastasis; N1: metastases in one to three axillary lymph nodes; N2: metastases in four to nine axillary lymph nodes; N3: metastases in ten or more axillary lymph nodes. Grade 1: well differentiated (low grade); Grade 2: moderately differentiated (intermediate grade); Grade 3: poorly differentiated (high grade); Grade 4: undifferentiated (high grade). ^∗^*P* < 0.05, ^∗∗^*P* < 0.01, ^∗∗∗^*P* < 0.001.

Firstly, we analyzed the expression levels of EIF4G2 in multiple tumors and normal tissues based on the TIMER2.0 database. As shown in Figure 1(a), compared with normal controls, EIF4G2 was markedly upregulated in a variety of cancers, including esophageal carcinoma (ESCA), cholangiocarcinoma (CHOL), glioblastoma multiforme (GBM), head and neck squamous cell carcinoma (HNSC), liver hepatocellular carcinoma (LIHC), lung squamous cell carcinoma (LUSC), and stomach adenocarcinoma (STAD). Then, the GEPIA database was used to validate the expression of EIF4G2. The results showed that EIF4G2 expression was significantly elevated in GC (Figure 1(b), *P* < 0.01). Subsequently, the association between EIF4G2 expression and clinicopathological features in GC patients was investigated using the UALCAN platform. As presented in Figures 1(c)–1(g), we found that based on the analysis of sample types, patients' gender, nodal metastasis status, individual cancer stages, and tumor grades, the expression of EIF4G2 in GC tissues was significantly higher than in normal tissues. All the above suggested that EIF4G2 was abnormally overexpressed in GC and could possibly serve as a biomarker of GC.

### 3.2. High Expression of EIF4G2 Indicated Poor Prognosis in GC

**Figure 2:**
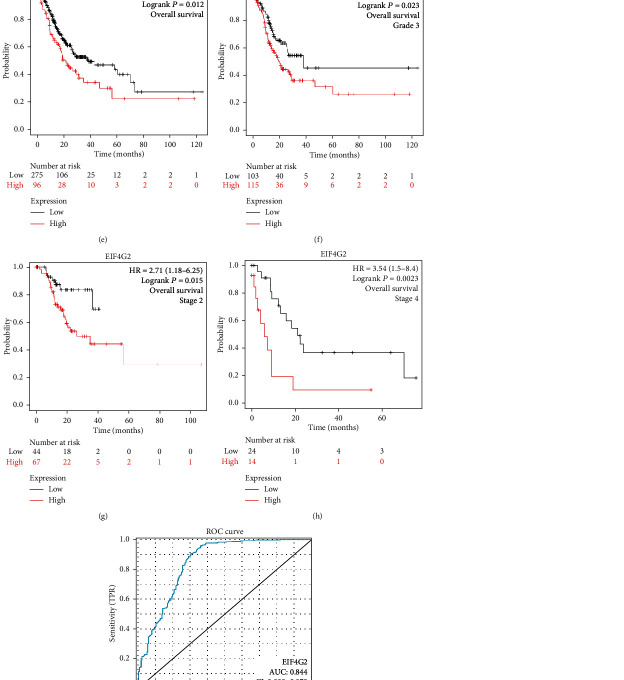
Prognostic significance and diagnostic value of EIF4G2 expression in GC. (a) The OS survival curves of GC in the GEPIA database. (b–h) Kaplan–Meier curves comparing high and low expression of EIF4G2 in gastric cancer based on gene chips and RNA-Seq data. (b–d) OS, PPS, and FP survival curves based on gene chips. (e–h) Survival curves based on RNA-Seq data; expression of EIF4G2 was significant overall (e), in grade 3 (f), stage 2 (g), and stage 4 (h) of gastric cancer patients' OS. (i) The diagnostic value of EIF4G2 in GC (ROC curve). Log-rank *P* < 0.05 was considered statistically significant with CI, confidence interval; FPR, false positive rate; TPR, true positive rate.

Next, survival analysis was performed to predict whether the expression of EIF4G2 affected GC patients' prognoses. As depicted in Figure 2(a), higher mRNA levels of EIF4G2 in GC were significantly associated with shorter overall survival (OS) in GEPIA. Then, the Kaplan–Meier plotter database was used to evaluate the prognosis associated with EIF4G2 expression in patients with GC, as well as the prognosis in different pathological subtypes. Figures 2(b)–2(d) shows that high expression of EIF4G2 was significantly correlated with poor OS, post progression survival (PPS) and first progression (FP) in GC. Exploiting the RNA-Seq data, we further verified the effect of EIF4G2 on the survival of GC patients. The results showed that high expression of EIF4G2 was negatively correlated with OS in patients with grade 3, stage 2, and stage 4 cancers (Figures 2(e)–2(h), *P* < 0.05). Finally, in view of its prognostic value in GC, we generated ROC curves to further analyze the diagnostic value of EIF4G2 in GC. As shown in Figure 2(i), the area under the curve (AUC) value was 0.844, indicating that EIF4G2 had good diagnostic ability to distinguish GC from normal controls. These results indicate that high expression of EIF4G2 is a biomarker of poor prognosis in GC, and that EIF4G2 may serve as a diagnostic biomarker for GC.

### 3.3. EIF4G2 Co-Expression Network in GC

**Figure 3:**
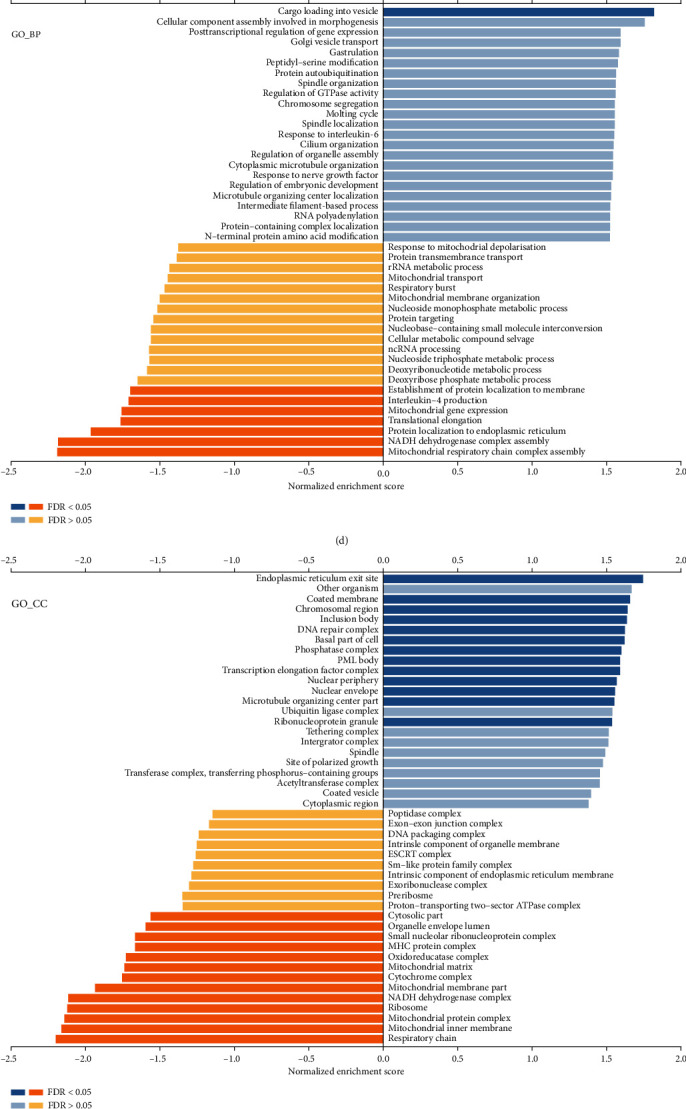
The co-expressed genes with EIF4G2 in GC from the LinkedOmics database. (a) All significantly associated genes with EIF4G2 distinguished by Pearson test in the GC cohort. (b–c) Heat maps showing the top 50 genes positively and negatively related to EIF4G2 in GC. Red represents positively linked genes and blue represents negatively linked genes. (d–g) GO analysis and KEGG pathways of the genes co-expressed with EIF4G2 in GC showing biological process (d), cellular component (e), molecular function (F) and KEGG pathways (g).

To investigate the mechanism of action for EIF4G2, the co-expression network of EIF4G2 was constructed using the LinkedOmics database. A volcano plot indicated that 11954 genes (dark red dots) were positively correlated with EIF4G2 expression, and 8271 genes (dark green dots) were negatively correlated (Figure 3(a)). The 50 genes with the strongest positive and negative correlations are presented in Figures 3(b)–3(c). Gene set enrichment analysis was then applied to analyze the GO terms and KEGG pathways of the genes co-expressed with EIF4G2. The results showed that at the GO-BP (biological process) level, these genes were mainly enriched in cargo loading into vesicle (Figure 3(d)). GO:CC (cellular component) was mainly involved in endoplasmic reticulum exit site, coated membrane and chromosomal region, among others (Figure 3(e)). GO:MF (molecular function) was mainly related to ubiquitinyl hydrolase activity, helicase activity, structural constituent of nuclear pore and regulatory RNA binding, among others (Figure 3(f)). KEGG pathway analysis indicated that the genes joined mainly in ubiquitin-mediated proteolysis, circadian rhythm, inositol phosphate metabolism, TGF-beta signaling pathway, and phosphatidylinositol signaling system (Figure 3(g)).

### 3.4. Correlation Analysis between EIF4G2 Expression and Immune Cell Infiltration in GC

**Figure 4:**
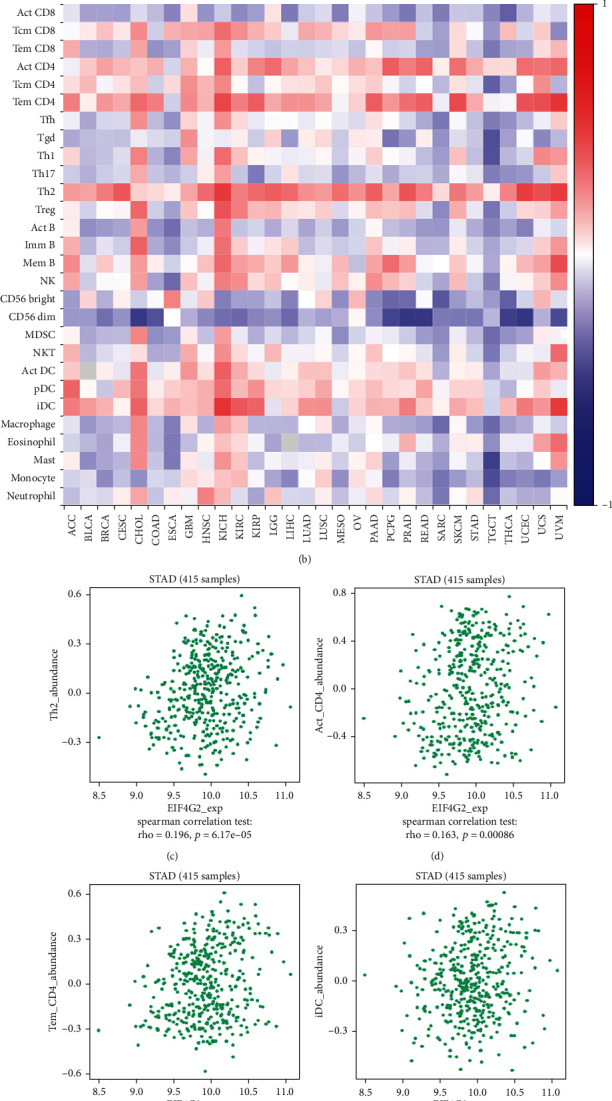
Correlations of EIF4G2 expression with immune infiltration level. (a) Correlations between expression of EIF4G2 with tumor purity, and infiltration level of B cells, CD4+ T cells, CD8+ T cells, neutrophils, macrophages and DCs in GC (TIMER2.0). (b) Relationship between the expression of EIF4G2 and 28 types of TILs across human cancers from TISIDB. (c–f) Top four TILs displaying the greatest Spearman correlation with EIF4G2 expression in GC. *P* < 0.05 is statistically significant.

Using TIMER 2.0, we analyzed the correlation between EIF4G2 expression and the six types of TIICs. As shown in Figure 4(a), EIF4G2 expression was significantly and positively associated with CD8+T cells (*r* = 0.257, *P* = 3.74*e* − 07), neutrophils (*r* = 0.283, *P* = 1.99*e* − 08), macrophages (*r* = 0.288, *P* = 1.19*e* − 08) and DCs (*r* = 0.138, *P* = 7.00*e* − 03) in GC. However, the results showed a negative correlation with infiltrating levels of B cells (*r* = −0.114, *P* = 2.68*e* − 02) and no correlation with CD4+ T cells (*r* = 0.026, *P* = 6.10*e* − 01). Then, we further evaluated the correlation between EIF4G2 expression and 28 types of TILs in the TISIDB database.  Figure 4(b) shows the relationship between expression of EIF4G2 and 28 types of TILs across human cancers. As presented in Figures 4(c)–4(f), the expression of EIF4G2 was correlated with abundance of Type 2 T helper cells (Th2; *r* = 0.196, *P* = 6.17*e* − 05), activated CD4 T cells (Act _CD4; *r* = 0.163, *P* = 0.00086), effector memory CD4 T cells (Tem _CD4; *r* = 0.164, *P* = 0.000832), and immature DCs (iDCs; *r* = 0.122, *P* = 0.013). These data indicated that EIF4G2 may play a specific role in immune infiltration in GC.

### 3.5. Correlation between EIF4G2 Expression and Immune Cell Markers in GC

Next, we further explored the relationship between EIF4G2 expression and TIIC markers in GC using the TIMER database. As shown in Table 1, we found that EIF4G2 was positively correlated with B cell markers (CD38), CD4 T Cell markers (CD4), M1 macrophage markers (NOS2, IRF5, PTGS2), M2 macrophage markers (CD163, VSIG4, MS4A4A, ARG1, MRC1), neutrophil markers (CEACAM8, ITGAM, CCR7, MPO), DC markers (NRP1, ITGAX, CD141), monocyte markers (CSF1R, CD86), natural killer cell markers (KIR2DS4, KIR3DL2, KIR3DL1, KIR2DL4, KIR2DL3, KIR2DL1), T cell markers (CD2), T cell exhaustion markers (CTLA4, HAVCR2), tumor-associated macrophage (TAM) markers (IL10), T follicular helper (Tfh) markers (BCL6, IL21), Th1 markers (TBX21, STAT4, IFNG), Th2 markers (STAT6, STAT5A), Th17 markers (STAT3) and T regulatory (Treg) markers (FOXP3, CCR8, STAT5B, TGFB1). The results showed that EIF4G2 could be involved in the regulation of tumor immune infiltration in GC.

### 3.6. Prediction and Analysis of Upstream miRNAs of EIF4G2

**Figure 5:**
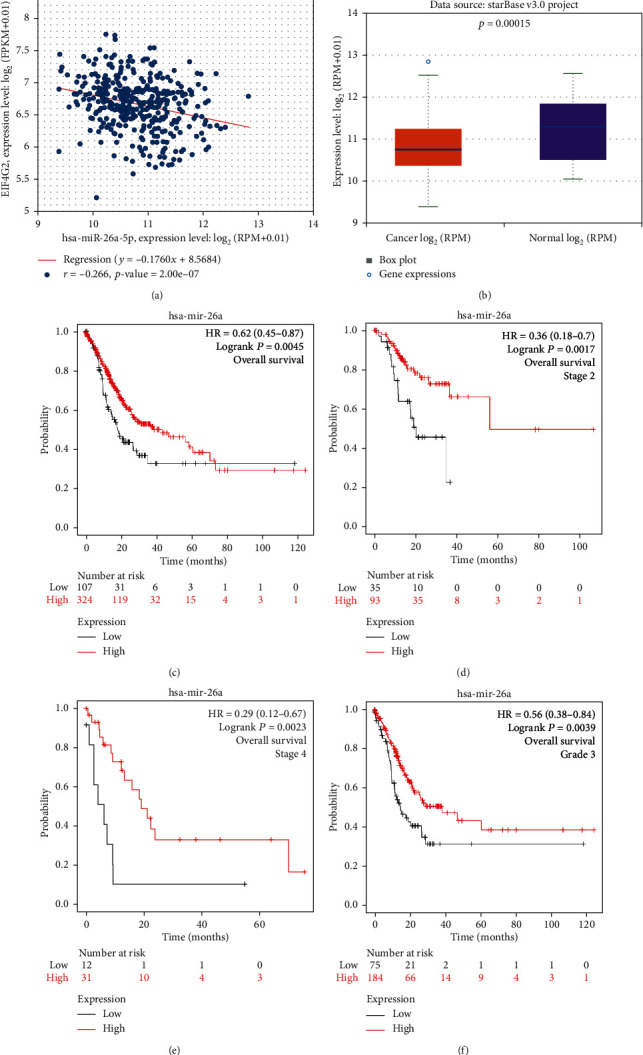
Identification of hsa-miR-26a-5p as a potential upstream miRNA of EIF4G2 in GC. (a) The expression correlation between hsa-miR-26a-5p and EIF4G2 in GC analyzed by the starBase database. (b) The expression of hsa-miR-26a-5p in GC and control normal samples determined by starBase. (c) OS survival curves of hsa-miR-26a-5p in GC. (d–f) Prognostic value of hsa-miR-26a-5p in GC patients with distinct clinicopathological statuses, including stage 2 (d), stage 4 (e), and grade 3 (f). *P* < 0.05 is statistically significant.

It has been widely acknowledged that noncoding (nc) RNAs participate in the regulation of gene expression. To ascertain whether EIF4G2 was modulated by some ncRNAs, we first predicted upstream miRNAs that could potentially bind to EIF4G2. Considering the underlying mechanisms of miRNAs in the regulation of target gene expression, we predicted a negative correlation between miRNAs and EIF4G2. Finally, we found 15 negatively correlated miRNAs (Table 2, *P* < 0.05) by starBase. Then, the expression level of these 15 miRNAs in GC and normal samples was examined. The miRNAs with low and statistically significant expression levels in GC patients were considered for analysis. Eventually, hsa-miR-26a-5p was identified. As shown in Figure 5(a), EIF4G2 was negatively correlated with hsa-miR-26a-5p, and only hsa-miR-26a-5p was significantly downregulated in GC (Figure 5(b), *P* < 0.001). Subsequently, the prognostic value of hsa-miR-26a-5p in GC was investigated. As described in Figures 5(c)–5(f), upregulation of hsa-miR-26a-5p was positively correlated with OS and with favorable OS in patients with stage 2, stage 4, and grade 3 cancers. These findings all suggested that hsa-miR-26a-5p may be the most likely regulatory miRNA of EIF4G2 in GC.

### 3.7. Prediction and Analysis of Upstream lncRNAs of EIF4G2

**Figure 6:**
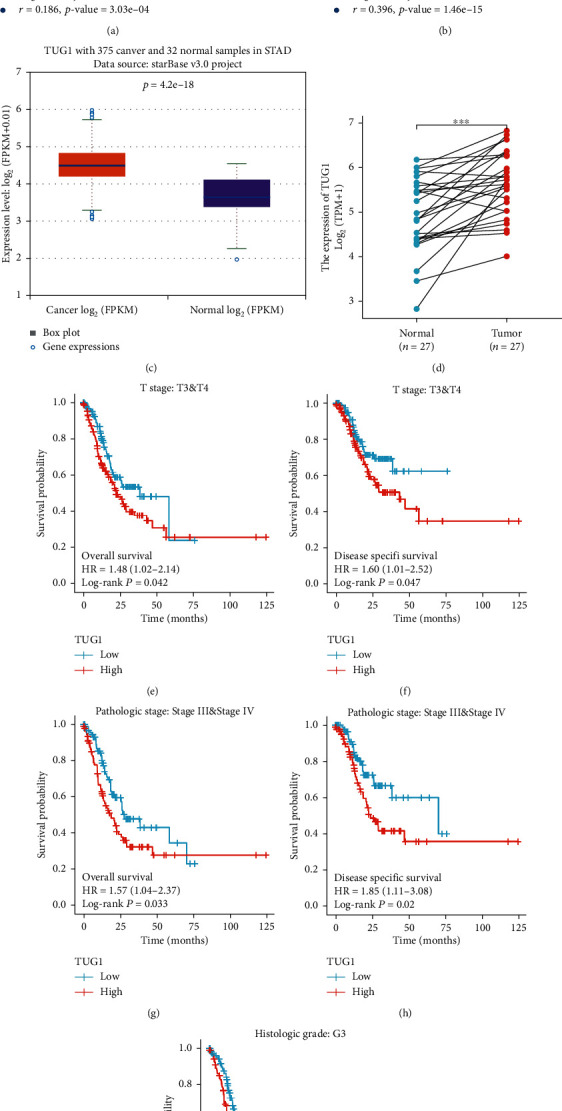
Analysis of TUG1 as the most likely upstream lncRNA of EIF4G2 in GC. (a) The expression correlation between hsa-miR-26a-5p and TUG1 in GC. (b) The expression correlation between EIF4G2 and TUG1 in GC. (c) The expression levels of TUG1 in 375 GC samples and 32 normal samples. (d) The expression levels of TUG1 in 27 GC and matched-adjacent normal samples. Survival curves show the association of TUG1 expression with OS and DSS in GC patients with different clinicopathological parameters: OS curve (e) and DSS curve (f) in T3 and T4 patients, OS curve (g) and DSS curve (h) in Stage III & Stage IV patients, OS curve (i) in grade 3 patients. ^∗∗∗^*P* < 0.001, ^∗^*P* < 0.05.

Next, the upstream lncRNAs of hsa-miR-26a-5p were predicted using the starBase database. According to the competing endogenous RNA (ceRNA) hypothesis, lncRNAs can increase mRNA expression by competitively binding to shared inhibitory miRNAs. Therefore, there should be negative correlation between lncRNAs and miRNAs and a positive correlation between lncRNAs and mRNAs. A total of nine possible lncRNAs were predicted (Tables 3–4, *P* < 0.05). Then, the expression levels and prognostic values of these lncRNAs in GC were assessed. The lncRNAs with high and statistically significant expression levels and unfavorable prognosis in GC patients were further analyzed. Ultimately, only TUG1 met the requirements. Figures 6(a)–6(b) shows the negative correlation between hsa-miR-26a-5p and TUG1 and the positive correlation between TUG1 and EIF4G2. As shown in Figures 6(c)–6(d), TUG1 was significantly upregulated in GC compared with normal controls. As described in Figures 6(e)–6(i), overexpressed TUG1 indicated poor OS or shorter disease-specific survival (DSS) of GC patients with different clinicopathological parameters. Taking expression analysis, survival analysis and correlation analysis into consideration, TUG1 appears to be the most likely upstream lncRNA regulating the hsa-miR-26a-5p/EIF4G2 axis in GC.

## 4. Discussion

GC is a malignant tumor with high incidence and poor prognosis worldwide. As we know, helicobacter pylori (HP) are closely associated with GC. Eradication of HP was suggested to be an effective method to reduce the risk of gastric carcinogenesis and treatment for GC. However, patient compliance, drug reactions, and resistance were considered as difficulties in the eradication therapy for HP infection [22]. Allium vegetables and their constituents were found to suppress gastric tumorigenesis and, thus, could as a therapeutic option for patients with GC. Additionally, Allium constituents were reported to prevent excessive HP growth and ultimately lower the risk of GC. Nevertheless, the results were controversial and required further validation [23]. Despite with a great progresses have been made in aspects of diagnosis, treatment, and prognosis, the outcome of GC patients remains unsatisfactory. Exploring the molecular mechanism of GC tumorigenesis may provide a new direction for identifying potential therapeutic targets and prognostic biomarkers. EIF4F is known as a protein complex which functions to regulate translation, composed of the cap-binding protein EIF4E, scaffolding protein EIF4G, and ATP-dependent RNA helicase EIF4A [24]. EIF4G2 (alias: death associated protein 5, DAP5; p97) is a subtype of EIF4G [25]. Many studies have reported that EIF4G2 is abnormally expressed in different cancers and has great influence on the progression of tumors. For instance, miR-139-5p suppresses aberrant protein translation by downregulating EIF4G2 expression in myeloid leukemia [26]. Leukemia/lymphoma-related factor interacts with DAP5 to inhibit p53 expression, resulting in tumor cell growth in colon cancer [27]. A recent study found that LINC01579 promotes cell proliferation by competitively binding with miR-139-5p to upregulate EIF4G2 in glioblastoma [28]. Another discovery confirmed that EIF4G2 is upregulated in HCC tissues, and high expression of EIF4G2 has been strongly correlated with worse prognosis in tumor patients [29]. However, the expression level and effect of EIF4G2 in GC are still unknown and deserve further investigation.

In our research, we first used multiple databases to analyze the expression level, survival, and prognosis of EIF4G2 in GC. The analysis showed that EIF4G2 was markedly upregulated in GC compared with normal controls. EIF4G2 expression in GC patients with different clinicopathological features revealed that the expression level was higher in patients with advanced tumors. Further survival analysis found that a high expression level of EIF4G2 was significantly correlated with poor OS, PPS, and FP in GC. Moreover, the increasing expression of EIF4G2 in higher tumor stages and grades also indicated shorter OS. ROC curves indicated that EIF4G2 had good diagnostic ability to distinguish GC from normal controls. Taken together, the results demonstrated that EIF4G2 may play a key role in the carcinogenesis and progression of GC and should be regarded as a prognostic and diagnostic biomarker.

We then developed a co-expression network for EIF4G2 in GC to explore its underlying mechanisms of action. The genes that positively or negatively correlated with EIF4G2 expression are shown in Figure 3. Some of these genes have been reported to be involved in the development and prognosis of GC. For example, a study suggested that COPI coat complex subunit beta 1 (COPB1) mRNA level was upregulated in GC and indicated poor OS, while COPB1 expression in GC was positively correlated with TILs, PD-L1 and CTLA4 [30]. Another study revealed that miR-204-5p inhibited GC cell proliferation via downregulation of USP47 and RAB22A [31]. Histone deacetylase 10, a gene negatively associated with EIF4G2 expression, was found to be downregulated in GC and was associated with an unfavorable prognosis [32]. Therefore, these findings further validated that the function of EIF4G2 in GC might partly involve interactions with these genes.

Next, through the database analysis, we observed that EIF4G2 expression was involved in tumor immune infiltration, and was positively associated with CD8+ T cell, neutrophil, macrophage, and dendritic cell infiltration levels. Additionally, the expression of EIF4G2 exhibited positive correlations with most biomarkers of TIICs, including those for macrophages, neutrophils, DCs, TAMs, and different T cell subsets. High infiltration of macrophages or TAMs has been reported to be associated with invasion and metastasis of GC, causing poor prognosis [33, 34]. Also, studies suggested that neutrophils could promote GC cell migration [35, 36]. Numerous reports about different functional T cell subsets have revealed that these immune cells contribute to tumor development and worse clinical outcomes [37–39]. These findings further confirm that EIF4G2 expression may be involved in the modulation of tumor immune infiltration in GC and influence the progression and prognosis of tumors.

An increasing number of studies have confirmed that ncRNAs such as miRNAs and lncRNAs are involved in the regulation of gene expression according to the ceRNA mechanism, in which lncRNA is regarded as an “miRNA sponge” that binds to inhibitory miRNAs targeting potential mRNAs and prevents their interaction with the mRNA, thus elevating the expression level of the targeted gene and in this way participate in the development of tumors [40–44]. In this study, we searched for upstream miRNAs of EIF4G2 by using starBase. Ultimately, hsa-miR-26a-5p was identified as a potential regulatory miRNA for EIF4G2 in GC. Our study found that hsa-miR-26a-5p was negatively correlated with EIF4G2 and was significantly downregulated in GC, and its low expression predicted unfavorable OS. In addition, patients with advanced cancer have a worse prognosis when hsa-miR-26a-5p expression is reduced. Several previous studies have demonstrated that the expression level of hsa-miR-26a-5p was decreased in multiple cancers, including colorectal cancer, bladder cancer, intrahepatic cholangiocarcinoma, and hepatocellular carcinoma, and also showed that low expression of hsa-miR-26a-5p was associated with tumor metastasis, leading to poor prognosis [45–49]. Based on the above studies we hypothesized that hsa-miR-26a-5p may be the most likely upstream miRNA of EIF4G2, modulating the functions of EIF4G2 in GC.

Next, the potential upstream lncRNAs of the hsa-miR-26a-5p/EIF4G2 axis were further investigated based on the ceRNA mechanism. The results showed that TUG1 was markedly upregulated in GC compared with normal controls, and the high expression level predicted poor prognosis, especially in higher cancer stages and grades. There have been many studies on the effect of TUG1 in tumors. For instance, a study reported that TUG1 was overexpressed in prostate cancer and promoted tumor cell migration, invasion, and proliferation by negatively modulating miR-26a expression [50]. Similarly, TUG1 was found to be highly expressed in colorectal cancer and facilitated the progression of cancer [51]. A positive role for TUG1 has also been discovered in pancreatic cancer, esophageal cancer, and ovarian cancer [52–54]. In recent years, TUG1 had been reported to act as a carcinogen in GC. Studies have pointed out that TUG1 was significantly overexpressed in GC and accelerated cell metastasis, invasion, and proliferation, resulting in poor prognosis [55–58]. These results indicate that the TUG1/hsa-miR-26a-5p/EIF4G2 axis is a novel regulatory pathway involved GC development.

## 5. Conclusion

In conclusion, we elucidated that EIF4G2 was upregulated in GC and showed that elevated expression of EIF4G2 indicated an unfavorable prognosis. Moreover, our findings showed that EIF4G2 expression may be involved in the modulation of tumor immune cell infiltration. In addition, we also assessed the potential upstream regulatory mechanisms of EIF4G2 in GC and found that the TUG1/hsa-miR-26a-5p/EIF4G2 axis was the most likely regulatory pathway. However, large-scale basic experiments and clinical trials are needed to validate these results. EIF4G2 is positively associated with the occurrence and development of GC and should be considered a prognosis biomarker and potential new therapeutic target.

## Figures and Tables

**Table 1:** Correlation analysis between EIF4G2 and markers of immune cells in GC.

Description	Gene marker	None		Purity	
		Cor	*P*	Partial. Cor	Partial. *P*
B cell	CD19	0.019	7.04E-01	0.015	7.75E-01
	CD38	0.196	6.20E-05	0.201	8.35E-05^∗∗∗^
CD8 T cell	CD8A	0.081	9.97E-02	0.088	8.57E-02
	CD8B	0.03	5.43E-01	0.046	3.69E-01
CD4 T cell	CD4	0.169	5.34E-04^∗∗∗^	0.179	4.64E-04^∗∗∗^
M1 macrophage	NOS2	0.086	8.06E-02	0.105	4.17E-02^∗^
	IRF5	0.116	1.86E-02^∗^	0.106	3.99E-02^∗^
	PTGS2	0.229	2.60E-06^∗∗∗^	0.242	1.91E-06^∗∗∗^
M2 macrophage	CD163	0.34	1.37E-12^∗∗∗^	0.347	3.64E-12^∗∗∗^
	VSIG4	0.169	5.63E-04^∗∗∗^	0.178	5.10E-04^∗∗∗^
	MS4A4A	0.213	1.28E-05^∗∗∗^	0.223	1.17E-05^∗∗∗^
	ARG1	0.139	4.63E-03^∗∗^	0.146	4.28E-03^∗∗^
	MRC1	0.319	3.51E-11^∗∗∗^	0.321	1.65E-10^∗∗∗^
Neutrophil	CEACAM8	0.14	4.16E-03^∗∗^	0.155	2.54E-03^∗∗^
	ITGAM	0.251	2.41E-07^∗∗∗^	0.252	6.63E-07^∗∗∗^
	CCR7	0.104	3.48E-02^∗^	0.12	1.91E-02^∗^
	MPO	0.191	9.35E-05^∗∗∗^	0.225	9.71E-06^∗∗∗^
DC	HLA-DRA	0.052	2.90E-01	0.057	2.67E-01
	HLA-DPA1	0.034	4.89E-01	0.034	5.05E-01
	NRP1	0.34	1.40E-12^∗∗∗^	0.342	7.89E-12^∗∗∗^
	ITGAX	0.232	2.00E-06^∗∗∗^	0.247	1.09E-06^∗∗∗^
	CD141	0.169	5.34E-04^∗∗∗^	0.165	1.23E-03^∗∗^
Monocyte	CSF1R	0.23	2.43E-06^∗∗∗^	0.231	5.47E-06^∗∗∗^
	CD86	0.194	6.68E-05^∗∗∗^	0.214	2.69E-05^∗∗∗^
NK cell	KIR2DS4	0.114	2.06E-02^∗^	0.12	1.96E-02^∗^
	KIR3DL3	0.032	5.11E-01	0.045	3.86E-01
	KIR3DL2	0.114	2.04E-02^∗^	0.132	1.03E-02^∗^
	KIR3DL1	0.147	2.74E-03^∗∗^	0.151	3.24E-03^∗∗^
	KIR2DL4	0.102	3.75E-02^∗^	0.112	2.91E-02^∗^
	KIR2DL3	0.163	8.89E-04^∗∗∗^	0.173	7.43E-04^∗∗∗^
	KIR2DL1	0.164	7.83E-04^∗∗∗^	0.189	2.12E-04^∗∗∗^
T cell	CD3D	0.022	6.51E-01	0.035	5.00E-01
	CD2	0.128	9.19E-03^∗∗^	0.145	4.73E-03^∗∗^
T cell exhaustion	CTLA4	0.209	1.91E-05^∗∗∗^	0.229	6.48E-06^∗∗∗^
	LAG3	0.063	2.02E-01	0.061	2.38E-01
	HAVCR2	0.211	1.53E-05^∗∗∗^	0.22	1.62E-05^∗∗∗^
	GZMB	0.057	2.43E-01	0.057	2.66E-01
	PDCD1	0.076	1.22E-01	0.093	7.16E-02
TAM	IL10	0.234	1.50E-06^∗∗∗^	0.246	1.24E-06^∗∗∗^
	CD68	0.109	2.67E-02^∗^	0.1	5.11E-02
Tfh	BCL6	0.238	9.36E-07^∗∗∗^	0.237	3.01E-06^∗∗∗^
	IL21	0.151	2.00E-03^∗∗^	0.155	2.46E-03^∗∗^
Th1	TBX21	0.097	4.83E-02^∗^	0.114	2.71E-02^∗^
	STAT4	0.249	3.14E-07^∗∗∗^	0.274	6.20E-08^∗∗∗^
	IFNG	0.163	8.73E-04^∗∗∗^	0.172	7.76E-04^∗∗∗^
	IL13	0.017	7.29E-01	0.024	6.48E-01
Th2	GATA3	-0.002	9.74E-01	0.024	6.48E-01
	STAT6	0.356	7.27E-14^∗∗∗^	0.353	1.35E-12^∗∗∗^
	STAT5A	0.321	2.10E-11^∗∗∗^	0.329	5.00E-11^∗∗∗^
Th17	STAT3	0.52	0.00E+00^∗∗∗^	0.517	2.68E-27^∗∗∗^
	IL17A	0.085	8.50E-02	0.084	1.03E-01
Treg	FOXP3	0.172	4.56E-04^∗∗∗^	0.185	2.85E-04^∗∗∗^
	CCR8	0.268	2.93E-08^∗∗∗^	0.284	1.73E-08^∗∗∗^
	STAT5B	0.447	0.00E+00^∗∗∗^	0.442	1.46E-19^∗∗∗^
	TGFB1	0.115	1.93E-02^∗^	0.113	2.76E-02^∗^

Cor, *R* value of Spearman's correlation; None, correlation without adjustment. Purity, correlation adjusted by purity. ^∗^*P* < 0.05, ^∗∗^*P* < 0.01, ^∗∗∗^*P* < 0.001.

**Table 2:** The predicted 15 miRNAs negatively correlated with EIF4G2 expression.

Gene	miRNA	*R* value	*P* value
EIF4G2	Hsa-let-7a-5p	-0.117	2.43E-02^∗^
EIF4G2	Hsa-let-7f-5p	-0.225	1.22E-05^∗∗∗^
EIF4G2	Hsa-miR-26a-5p	-0.266	2.00E-07^∗∗∗^
EIF4G2	Hsa-miR-26b-5p	-0.137	8.35E-03^∗∗^
EIF4G2	Hsa-miR-32-5p	-0.113	2.95E-02^∗^
EIF4G2	Hsa-miR-101-3p	-0.118	2.32E-02^∗^
EIF4G2	Hsa-let-7 g-5p	-0.156	2.60E-03^∗∗^
EIF4G2	Hsa-miR-30b-5p	-0.12	2.10E-02^∗^
EIF4G2	Hsa-miR-106b-5p	-0.102	4.99E-02^∗^
EIF4G2	Hsa-miR-374a-5p	-0.116	2.49E-02^∗^
EIF4G2	Hsa-miR-493-3p	-0.115	2.69E-02^∗^
EIF4G2	Hsa-miR-411-5p	-0.138	7.67E-03^∗∗^
EIF4G2	Hsa-miR-758-3p	-0.119	2.20E-02^∗^
EIF4G2	Hsa-miR-340-5p	-0.164	1.47E-03^∗∗^
EIF4G2	Hsa-miR-374b-5p	-0.181	4.43E-04^∗∗∗^

∗p < 0.05, ∗∗p < 0.01, ∗∗∗p < 0.001.

**Table 3:** The predicted nine lncRNAs negatively correlated with hsa-miR-26a-5p.

miRNA	lncRNA	*R* value	*P* value
Hsa-miR-26a-5p	NORAD	-0.2	1.04E-04^∗∗∗^
Hsa-miR-26a-5p	AC068768.1	-0.156	2.56E-03^∗∗^
Hsa-miR-26a-5p	TUG1	-0.186	3.03E-04^∗∗∗^
Hsa-miR-26a-5p	AC005261.1	-0.138	7.61E-03^∗∗^
Hsa-miR-26a-5p	OIP5-AS1	-0.146	4.64E-03^∗∗^
Hsa-miR-26a-5p	AC000120.1	-0.124	1.71E-02^∗^
Hsa-miR-26a-5p	AL139407.1	-0.194	1.72E-04^∗∗∗^
Hsa-miR-26a-5p	AC093297.2	-0.169	1.10E-03^∗∗^
Hsa-miR-26a-5p	EBLN3P	-0.172	8.82E-04^∗∗∗^

**Table 4:** The predicted nine lncRNAs positively correlated with EIF4G2.

lncRNA	mRNA	*R* value	*P* value
NORAD	EIF4G2	0.178	5.35E-04^∗∗∗^
AC068768.1	EIF4G2	0.237	3.46E-06^∗∗∗^
TUG1	EIF4G2	0.396	1.46E-15^∗∗∗^
AC005261.1	EIF4G2	0.133	9.78E-03^∗∗^
OIP5-AS1	EIF4G2	0.19	2.14E-04^∗∗∗^
AC000120.1	EIF4G2	0.178	5.27E-04^∗∗∗^
AL139407.1	EIF4G2	0.214	3.02E-05^∗∗∗^
AC093297.2	EIF4G2	0.129	1.26E-02^∗^
EBLN3P	EIF4G2	0.33	5.98E-11^∗∗∗^

∗P < 0.05, ∗∗P < 0.01, ∗∗∗P < 0.001.

## Data Availability

The datasets used and/or analyzed during the current study are available from the corresponding author on reasonable request. The data were obtained with these following databaases: TIMER2.0(http://timer.cistrome.org/); GEPIA(http://gepia.cancer-pku.cn/); UALCAN(http://ualcan.path.uab.edu/); KM plotter (http://kmplot.com/analysis/index.php?p=background); LinkedOmics (http://www.linkedomics.org/login.php); TISIDB (http://cis.hku.hk/TISIDB/); and starBase(https://starbase.sysu.edu.cn/).

## Abbreviations

EIF4G2: Eukaryotic translation initiation factor 4 gamma 2
GC: Gastric cancer
ROC: Receiver operator characteristic
TUG1: Taurine upregulated 1
eIFs: Eukaryotic translation initiation factors
DLBCL: Diffuse large B cell lymphoma
TIMER2.0: Tumor Immune Estimation Resource 2.0
GEPIA: Gene Expression Profiling Interactive Analysis
TISIDB: Tumor-Immune System Interaction Database
miRNAs: microRNAs
lncRNAs: Long noncoding RNAs
TIICs: Tumor-infiltrating immune cells
DCs: Dendritic cells
TCGA: The cancer genome atlas
GTEx: Genotype-tissue expression
KM plotter: Kaplan–Meier plotter
GEO: Gene expression omnibus
RNA-Seq: RNA sequencing
GO: Gene Ontology
KEGG: Kyoto Encyclopedia Of Genes And Genomes
TILs: Tumor-infiltrating lymphocytes
TPM: Transcripts per million
OS: Overall survival
PPS: Postprogression survival
FP: First progression
AUC: Area under the curve
BP: Biological process
CC: Cellular component
MF: Molecular function
TAM: Tumor-associated macrophage
ceRNA: Competing endogenous RNA
DSS: Disease-specific survival
DAP5: Death associated protein 5
COPB1: COPI coat complex subunit beta 1.
